# Epilepsy and TSC-Associated Neuropsychiatric Disorders

**DOI:** 10.15844/pedneurbriefs-34-11

**Published:** 2020-12-02

**Authors:** Jamie K. Capal

**Affiliations:** 1 Department of Neurology, Cincinnati Children’s Hospital Medical Center, Cincinnati, OH; 2 University of Cincinnati College of Medicine, Cincinnati, OH

**Keywords:** Tuberous Sclerosis Complex, TSC-Associated Neuropsychiatric Disorder, Autism Spectrum Disorder, Epilepsy

## Abstract

Investigators from multiple institutions (Cleveland Clinic, University Hospital Leuven, ZOL Genk, Kempenhaeghe and Maastricht UMC+, and Children’s Hospitals and Clinics of Minnesota) used the Tuberous Sclerosis Complex Natural History Database (TSCNHD) to evaluate the relationship between epilepsy and neuropsychiatric disorders in individuals with TSC, along with any potential influence of genotype.

Investigators from multiple institutions (Cleveland Clinic, University Hospital Leuven, ZOL Genk, Kempenhaeghe and Maastricht UMC+, and Children’s Hospitals and Clinics of Minnesota) used the Tuberous Sclerosis Complex Natural History Database (TSCNHD) to evaluate the relationship between epilepsy and neuropsychiatric disorders in individuals with TSC, along with any potential influence of genotype. Epilepsy was seen in 88% of patients and was more frequent in individuals with a TSC2 mutation. Individuals with TSC2 mutation were also more likely to have epilepsy onset at less than two years of age, as well as infantile spasms. Epilepsy was associated with intellectual disability (ID), particularly those with more severe ID. Also, age of seizure onset before age two years was associated with more severe ID but not epilepsy duration. Autism spectrum disorder (ASD) was diagnosed more frequently in patients with co-existing epilepsy, particularly among those with an age of seizure onset before two years. There was a trend toward a higher frequency of ID and ASD in individuals with a TSC2 mutation. There was no association with genotype. ADHD was diagnosed more frequently in patients with co-morbid epilepsy. Neither anxiety nor depression was associated with epilepsy or genotype. The study concludes that Epilepsy is associated with an increased risk for intellectual disability, autism spectrum disorder, and ADHD in individuals with TSC. Earlier age of seizure onset appears to increase the risk for several neuropsychiatric disorders in TSC. [1]

COMMENTARY. Tuberous Sclerosis Complex (TSC) is a genetic disorder with a high incidence of epilepsy and TAND (TSC-Associated Neuropsychiatric Disorders). The study showed a strong association between early onset of epilepsy and intellectual disability, and risk for development of ASD. This finding is consistent with several prospective studies, including a prospective study by Bolton and colleagues, who found that a higher tuber load, history of status epilepticus, and genetic mutation for TSC contributed to overall cognition [2]. A large, multi-center, prospective natural history study of young children with TSC found that early onset of seizures was associated with worse developmental outcomes and a higher risk of developing ASD [3,4]. A significant strength of the current study was utilizing the TSC Natural History Database, which was established to characterize and study many individuals with TSC throughout the lifespan. The relationship between early seizures and subsequent TAND highlights the need for more early preventative treatment.

## Disclosures

The author has declared that no competing interests exist.
